# Validity and timeliness of syndromic influenza surveillance during the autumn/winter wave of A (H1N1) influenza 2009: results of emergency medical dispatch, ambulance and emergency department data from three European regions

**DOI:** 10.1186/1471-2458-13-905

**Published:** 2013-10-01

**Authors:** Nicole Rosenkötter, Alexandra Ziemann, Luis Garcia-Castrillo Riesgo, Jean Bernard Gillet, Gernot Vergeiner, Thomas Krafft, Helmut Brand

**Affiliations:** 1 Department of International Health, CAPHRI School for Public Health and Primary Care, Faculty for Health, Medicine and Life Sciences, Maastricht University, Duboisdomein 30, Maastricht 6229 GT, The Netherlands; 2 Department of Medical Sciences and Surgery, University of Cantabria, Santander, Spain; 3 Department of Emergency Medicine, University Hospital Leuven, Leuven, Belgium; 4 Dispatch Centre Tyrol, Innsbruck, Austria

**Keywords:** Public health surveillance, Syndromic surveillance, Influenza, Emergency medical service, Sensitivity, Specificity, Timeliness

## Abstract

**Background:**

Emergency medical service (EMS) data, particularly from the emergency department (ED), is a common source of information for syndromic surveillance. However, the entire EMS chain, consists of both out-of-hospital and in-hospital services. Differences in validity and timeliness across these data sources so far have not been studied. Neither have the differences in validity and timeliness of this data from different European countries. In this paper we examine the validity and timeliness of the entire chain of EMS data sources from three European regions for common syndromic influenza surveillance during the A(H1N1) influenza pandemic in 2009.

**Methods:**

We gathered local, regional, or national information on influenza-like illness (ILI) or respiratory syndrome from an Austrian Emergency Medical Dispatch Service (EMD-AT), an Austrian and Belgian ambulance services (EP-AT, EP-BE) and from a Belgian and Spanish emergency department (ED-BE, ED-ES). We examined the timeliness of the EMS data in identifying the beginning of the autumn/winter wave of pandemic A(H1N1) influenza as compared to the reference data. Additionally, we determined the sensitivity and specificity of an aberration detection algorithm (Poisson CUSUM) in EMS data sources for detecting the autumn/winter wave of the A(H1N1) influenza pandemic.

**Results:**

The ED-ES data demonstrated the most favourable validity, followed by the ED-BE data. The beginning of the autumn/winter wave of pandemic A(H1N1) influenza was identified eight days in advance in ED-BE data. The EP data performed stronger in data sets for large catchment areas (EP-BE) and identified the beginning of the autumn/winter wave almost at the same time as the reference data (time lag +2 days). EMD data exhibited timely identification of the autumn/winter wave of A(H1N1) but demonstrated weak validity measures.

**Conclusions:**

In this study ED data exhibited the most favourable performance in terms of validity and timeliness for syndromic influenza surveillance, along with EP data for large catchment areas. For the other data sources performance assessment delivered no clear results. The study shows that routinely collected data from EMS providers can augment and enhance public health surveillance of influenza by providing information during health crises in which such information must be both timely and readily obtainable.

## Background

Influenza surveillance systems monitor the occurrence and progress of the disease so as to support influenza management during epidemics. Clinical and virological influenza surveillance systems have been established in the European member states [1,2], and the European Centre for Disease Prevention and Control (ECDC) aggregates data regarding influenza occurrence from these systems to enhance monitoring and reporting of disease trends across Europe [3].

Syndromic surveillance systems based on immediate, usually electronically available, routine health information are increasingly being added to traditional surveillance structures (i.e., clinical / sentinel or virological) to establish more comprehensive surveillance or epidemic intelligence systems [4,5]. Typically based on the use of existing routine data, the systems do not require new data collection mechanisms. However, since the data are not being collected primarily for surveillance purposes, the provided information covers only signs and symptoms and contains no clinically verified or laboratory-confirmed diagnoses [5]. Due to real-time or near real-time data availability, syndromic surveillance systems are designed to enhance the identification of immediately occurring or out-of-season health threats, such as pandemic influenza. Existing syndromic surveillance approaches apply indicator-based components, such as data from emergency departments [6,7], emergency medical dispatch centres [8,9], and telephone help lines [10,11]; as well as information on school-absenteeism [12,13] or over-the-counter drug sales of analgesics [14]. The data may be even broader, systems that apply event-based information use information from media sources or web queries related to influenza [15,16].

European and international syndromic surveillance systems based on event-based health information exist. The Directorate General for Health and Consumers of the European Commission (EC), for example, directs the Medical Information System (MedISys), which monitors the international media for general disease occurrence information but also specifically for influenza activity [17]. Routine syndromic surveillance systems based on indicator-based components, however, are scarce and are, at least in Europe, the individual efforts of single regions or countries. A European study to identify commonalities and good practice in national or regional syndromic surveillance activities has been lacking for a long time and has now been established by an EC co-founded project [18]. The analysis of the potential for a European-wide application of emergency medical service (EMS) data for indicator-based syndromic influenza surveillance is missing so far [19].

Moreover, existing national and regional EMS data-based syndromic surveillance systems do not focus on the entire chain of available data. Data covering the entire EMS chain consists of out-of-hospital emergency medical dispatch (EMD) information on signs and symptoms typically described by laypeople calling for an ambulance; ambulance service (EP) data on the initial diagnostic findings during examination at the emergency scene by paramedics or emergency physicians; and in-hospital information from nurses or physicians at the emergency department (ED) covering the patient’s main complaints or the initial diagnostic findings during the patient’s treatment in the ED [20]. Typically, however, EMS data-based syndromic influenza surveillance systems focus mostly on ED data, only a few include data from the EMD, and to our knowledge, EP data is not yet exploited by any syndromic influenza surveillance system. Thus, little is known about the differences in the performance of syndromic influenza surveillance based on the three levels of available emergency medical service data and the applicability of this health information for syndromic influenza surveillance in various European countries.

To evaluate the performance of a common syndromic influenza surveillance approach based on the EMD, EP and ED data from different European regions during the autumn/winter wave of the A(H1N1) influenza pandemic, we focus on the validity components, sensitivity and specificity, as well as on timeliness measures as described by Buehler et al. [21]. The validity and timeliness assessment is performed retrospectively against traditional influenza surveillance sources.

## Methods

### Time period of the analysis

In Europe, the autumn/winter wave of the pandemic A(H1N1) influenza began around week 43 of 2009, earlier than the beginning of the normal seasonal influenza cycle. The ECDC registered the modal peak of the autumn/winter wave at approximately week 48 in Europe [22,23]. In this study, the validity and timeliness of syndromic surveillance data were assessed during the time period between week 36 (start 30.8.) to week 52 (end 31.12.) in 2009 (N = 17 weeks; N = 123 days). Due to limited data availability, the first weeks of 2010 were not analysed. However, as reported by ECDC, most of the disease burden in regard to the pandemic A(H1N1) influenza occurred by the end of 2009 [22,23].

### Data sets

#### Syndromic surveillance data

Data for this study were retrieved during the SIDARTHa project on emergency data-based syndromic surveillance [24]. The SIDARTHa project group consisted of EMS institutions from 12 European countries. Three partner institutions, designated as test sites and consisting of EMD centres, ambulance services (EP), or EDs delivered in total five data sets from a local, regional, or national level for this study. The number of specific EMS data sources per country used in this study is not related to the general availability of these data in Europe.

The city- or district-level data sources included ambulance service data for the district of Kufstein in Austria (EP-AT) and emergency department data for the city of Leuven in Belgium (ED-BE) and Santander in Spain (ED-ES). Regional emergency call data were provided by the Dispatch Centre Tyrol in Austria (EMD-AT), which at that time covered three out of nine districts in Tyrol. The ambulance service of Belgium (EP-BE) provided national data. For the sake of readability, we refer in the following text to the composite abbreviations of each data source (e.g., EMD-AT), including information on the respective emergency medical service (e.g., EMD for Emergency Medical Dispatch) and the country code (e.g., AT for Austria). The country code does not imply that the data sources are representative for the whole countries. More specific information on the properties of each data set can be found in Table 1.

**Table 1:** Properties of the syndromic surveillance data sets

		**Geographical specification of the data source**					
**Abbreviation of the data source**	**Data source**	**Country**	**Region**	**City/District**	**Population served (approx.)**	**Data provider**	Baseline period*
EMD-AT	Emergency Medical Dispatch	Austria	Tyrol data source covers 3 of 9 districts in Tyrol	Innsbruck city, Innsbruck district, Kufstein district	380,000	Dispatch Centre Tyrol	1/2005-12/2008
							Except cases from December to March each year.
EP-AT	Emergency Physician service (ambulances)	Austria	Tyrol	Kufstein district	99,000	Dispatch Centre Tyrol	1/2006-12/2008
							Except cases from November to March each year.
EP-BE	Emergency Physician service (ambulances)	Belgium			10,500,000	Ministry of Health, Belgium	3/2009-8/2009
ED-BE	Emergency Department	Belgium	Flemish Brabant	Leuven	a. 91,000 (Leuven) b. 1,000,000 (reference hospital for the region Flemish Brabant)	University Hospital Leuven	3/2009-8/2009
ED-ES	Emergency Department	Spain	Autonomous Region Cantabria	Santander	a. 300,000 (Santander) b. 580,000 (reference hospital for the Autonomous Region Cantabria)	University Hospital Marqués de Valdecilla	8/7/2009-30/8/2009

*Baseline period = period which was used for the adjustment of the aberration detection algorithm (Poisson CUSUM).

All data sets included anonymous health information on individual patients who sought the respective EMS. The data were available on a daily scale.

#### Reference data

Reference data were retrieved from regional or national clinical (sentinel) influenza surveillance systems. The data included weekly reports from physicians, usually general practitioners (GP), regarding the number of patients treated for ILI and were suitable to assess the course and spatial distribution of influenza [23]. Since the autumn/winter wave of the A(H1N1) influenza pandemic 2009 began sooner than the normal seasonal cycle, the Austrian sentinel system for the Tyrol region was not active. As a substitute, data on the number of documented sick-leave cases with acute respiratory illness (ARI) were retrieved from a major Tyrolean health insurance (Tiroler Gebietskrankenkasse). This health insurance covers approximately 75% of the Tyrolean population [25].

The reference data included weekly case numbers registered at the time of the case occurrence. The properties of the respective reference data sources are given in Table 2. The table also includes information on the reporting delay between case occurrence and data availability at the respective public health authority.

**Table 2:** Properties of the reference data

				**Influenza pandemic according to reference data in 2009**			
**Reference data for…**	**Reference data source**	**Geographical level**	Reporting delay*	**Start week**	**Peak week**	**Duration (weeks)**	**Duration (days)**
EMD-AT^†^, EP-AT^†^	Information on sick leave due to acute respiratory infections from a major Tyrolean health insurance.	regional	information for week x available on week x + 1	44	47	9	67^$^
		(Tyrol, Austria)					
EP-BE,	Notified influenza cases of the sentinel general practitioner system.	national	information for week x available on week x + 3	40	44	10	70
ED-BE		(Belgium)					
ED-ES	Notified influenza cases of the sentinel general practitioner system.	regional	information for week x available on week x + 3	41 (period 1)	43	8	56
		(Autonomous Region Cantabria, Spain)		49 (period 2)			

*week: Monday (day 1), …, Sunday (day 7).

^†^The course of the autumn/winter wave was derived from regional data of the Tyrolean health insurance; the start week of the autumn/winter wave was derived from national data [27].

^$^last week of 2009 (week 52) contained 11 days.

The onset of the A(H1N1) influenza pandemic was determined by pre-defined thresholds as specified by the respective public health authorities: The Belgian reference data, defined the threshold as more than 141.37 ILI cases per 100,000 inhabitants treated by sentinel GPs per week [26], while the Spanish sentinel system for the Autonomous Region of Cantabria determined a threshold at more than 71 ILI cases per 100,000 inhabitants in GP practices per week. Case occurrence of less than 71 ILI cases per 100.000 inhabitants resulted in a temporary cessation in the epidemic period in 2009 (week 48) in the Autonomous Region of Cantabria. Since no threshold was determined for the Austrian reference data, we applied the official national reported beginning of the A(H1N1) pandemic in Austria, which was based on the number of laboratory-confirmed A/H1N1 influenza cases. In this report, a reference on the determination of the beginning of the epidemic (e.g., a predefined threshold) is missing [27]. A summary of the reference data properties regarding the autumn/winter wave is exhibited in Table 2.

### Variables

The main variables were the date of the emergency occurrence and the information on the health status of the emergency cases. The day on which the emergency case occurred was used to identify the day-of-the-week variation in the data sets.

The European Influenza Surveillance Network defines relevant health information for ILI for clinical surveillance and recommends a combination of influenza symptoms as an ILI case definition [28]. Since the study presented in this paper is based on routine information from EMS providers, it could use only a set of single pre-defined major symptoms reported by the emergency caller, or chief complaints or a working diagnosis identified during the admission at the ED or provided by the ambulance staff at the emergency scene. As identified in previous studies, these broad-symptom categories or working diagnoses exhibit a moderate sensitivity in meeting a clinically confirmed influenza diagnosis [29] or correspondence to the epidemic curves of the clinical sentinel surveillance system [30].

For the respective data sets in this study, health information was available as single codes from the Advanced Medical Priority Dispatch System (AMPDS [EMD-AT]), the International Classification of Diseases ((ICD-9 [EP-BE]; ICD-10 [EP-AT]), free-text information regarding the chief complaint and/or the working diagnosis (ED-BE), and regional chief complaint triage codes (ED-ES) (Table 3).

**Table 3:** Health information used for respiratory syndrome and influenza-like illness coding and respective code distribution in 2009

**Data source**	**Syndrome**	**Health information codes**		**Code distribution 2009**	
EMD-AT	respiratory syndrome	AMPDS v12.0	Boolean operator : OR	N	%
		6C1	Breathing problems - Abnormal breathing	465	26.9
		6C1A	Breathing problems - abnormal breathing + asthma	71	4.1
		6D1	Breathing problems - Not alerting	750	43.3
		6D1A	Breathing problems - not alerting + asthma	230	13.3
		6D2	Breathing problems - Difficulty in speaking between breaths	22	1.3
		6D2A	Breathing problems - difficulty in speaking between breaths + asthma	4	0.2
		6D3	Breathing problems - changing colour	154	8.9
		6D3A	Breathing problems - changing colour + asthma	31	1.8
		6D4	Breathing problems - clammy	-	-
		6D4A	Breathing problems - clammy + asthma	-	-
		26A4	Sick person - Fever/chills	4	0.2
		26O26	Sick person - Sore throat (without difficulty breathing or swallowing)	-	-
**Total**				**1731**	**100.0**
**EP-AT**	ILI*	ICD-10	Boolean operator : OR		
		J00	Acute nasopharyngitis [common cold]	1	1.6
		J02	Acute pharyngitis (includes sore throat)	-	-
		J04	Acute laryngitis and tracheitis	3	4.7
		J06	Acute upper respiratory infections of multiple and unspecified sites	8	12.5
		J09	Avian Influenza	-	-
		J10	Influenza due to other identified influenza virus	1	1.6
		J11	Influenza, virus not identified	8	12.5
		J16	Pneumonia due to other infectious organisms, not elsewhere classified	3	4.7
		J18	Pneumonia, organism unspecified	28	43.8
		R05	Cough	3	4.7
		R50	Fever of other and unknown origin	9	14.1
**Total**				**64**	**100.0**
**EP-BE**	ILI*	ICD-9	Boolean operator : OR		
		460	Acute nasopharyngitis [common cold]	22	1.2
		462	Pharyngitis, acute	-	-
		464	Acute laryngitis and tracheitis	-	-
		464.0	Acute laryngitis	-	-
		464.1	Acute tracheitis	-	-
		464.2	Acute laryngotracheitis	-	-
		465	Acute upper respiratory infections of multiple or unspecified sites	-	-
		465.0	Acute laryngopharyngitis	-	-
		465.8	Acute upper respiratory infections of other multiple sites	-	-
		465.9	Acute upper respiratory infections of unspecified site	-	-
		480.9	Viral pneumonia unspecified	56	3.0
		488	Influenza due to certain identified influenza viruses	-	-
		488.0	Influenza due to identified avian influenza virus	-	-
		488.1	Influenza due to identified novel h1n1 influenza virus	-	-
		487	Influenza	-	-
		487.0	Influenza with pneumonia	-	-
		487.1	Influenza with other respiratory manifestations	-	-
		487.8	Influenza with other manifestations	109	5.9
		486	Pneumonia organism unspecified	986	53.2
		786.2	Cough	63	3.4
		780.6	Fever and other physiologic disturbances of temperature regulation	618	33.3
**Total**				**1854**	**100.0**
**ED-BE**	ILI*	chief complaint or working diagnosis, Boolean operator : OR			
	free text including		cough, muscle pain, flu, H1N1, sore throat, influenza, fever	na^†^	na^†^
**Total**				**5681**	**100.0**
**ED-ES**	ILI*	case definition, Boolean operator : AND			
			i) the appearance of sudden symptoms and at least one of the four general symptoms (fever or slight fever (feverishness), headache, muscle pain, general malaise), and (ii) at least one of the three respiratory symptoms (cough, sore throat, difficulty breathing), as well as (iii) the absence of other diagnostic suspicion.	1127	100.0
**Total**^**$**^				**1127**	**100.0**

*ILI = influenza-like illness.

^†^na = not applicable.

Relevant codes for monitoring ILI were defined for each EMS coding system based on available literature and the expertise of EMS experts from the SIDARTHa consortium (Table 3). Since the health information derived from the AMPDS codes (EMD-AT) was not specific enough to differentiate between respiratory syndrome and ILI, we analysed the respiratory syndrome in this data set. In the ED-ES data, the ILI case definition was designed as a fixed list of combined chief-complaint triage codes comparable to the ILI definition contained in the reference data set of the Spanish sentinel surveillance system (Autonomous Region Cantabria) (Table 3).

The share of AMPDS, ICD-9 or ICD-10 codes presented in Table 3 indicates the structure of ILI or respiratory syndrome in the syndromic surveillance data sources. In the EMD-AT data, respiratory syndrome cases were coded primarily as severe breathing problems. ILI cases in ICD-coded data sets (EP-AT, EP-BE) mostly received a working diagnosis of pneumonia or fever. The exploitation of a broad range of free text items, which allowed different writings and short forms, made it impossible to describe the structure of ILI in ED-BE data.

Cases to which respiratory syndrome or ILI was assigned were aggregated per week and per day for further analysis.

### Statistical analysis

#### Characteristics of syndromic surveillance data

The characteristics of the individual syndromic surveillance data sources during the respective baseline period and the test period (week 36/2009 to week 52/2009) were analysed using general descriptive statistics. The selection of suitable baseline periods for the individual data sources (Table 1) was driven by data availability and a thorough descriptive analysis of variations in daily case numbers per year and per month to ensure stability (reported elsewhere [31]). Due to comprehensive data availability in the Austrian data sets (EMD-AT and EP-AT), we were able to exclude the spring and summer period of 2009, during which the 2009 influenza pandemic started, from the baseline period of these data sets. In the other data sets, limited data availability led to the inclusion of these periods. Additionally, day-of-the-week variation was analysed in the baseline data sets employing Kruskal-Wallis test statistics (significance level p < 0.05).

#### Aberration detection

Aberrations in the daily number of patients with respiratory syndrome or ILI during the test period (week 36/2009 to week 52/2009) were investigated using a one-sided cumulative sum (CUSUM) aberration detection algorithm for Poisson-distributed data [32] in combination with the Fast Initial Response (FIR) mechanism [32,33]. The FIR technique ensures that large CUSUM values do not inflate subsequent values, thus controlling for an over-production of signals. It also allows a head start of the algorithm to retrieve quicker signals [33]. The Poisson CUSUM algorithm is based on the individual baseline mean from which the reference value *k*, the head start value *S*_*0*_*,* and the threshold value *h* are determined.

More specifically the reference value *k* was determined by the following equation:

$$k=\left(\mu_{d}-\mu_{a}\right)/\left(\ln\left(\mu_{d}\right)-\ln\left(\mu_{a}\right)\right)$$

The acceptable process mean (*μ*_*a*_) was set close to the baseline mean (*μ*_*d*_) as described by Lucas [34]. When *k* was larger or equal to one, the value was rounded to the nearest integer.

The daily Poisson CUSUM value was calculated as follows [34]:

$$S_{H,i}=\max\left(0,Y_{i}-k+S_{H,i-1}\right)$$

The threshold value *h* for the CUSUM algorithm and the head start value *S*_*0*_ were retrieved from a table provided by Lucas [34]. *Y*_*i*_ represented the daily number of respiratory syndrome or ILI cases. A signal was produced whenever the daily CUSUM value *S*_*H,i*_ was greater than or equal to the respective threshold value *h*, indicating a significant change in the time series. The respective set-ups and threshold values for the Poisson CUSUM algorithm per data set are listed in Table 4.

**Table 4:** Characteristics of the daily number of respiratory syndrome or influenza-like illness cases during baseline and the test period (week 36 to week 52, 2009), test statistics on the probability distribution of daily counts, the identification of day-of-the-week effects, and Poisson CUSUM parameters during individual baseline periods

	**EMD-AT**		**EP-AT**		**EP-BE**		**ED-BE**		**ED-ES**	
	**Respiratory syndrome**		**ILI**		**ILI**		**ILI**		**ILI**	
**Period**	Baseline^*^	**Test**^**†**^	Baseline^*^	**Test**^**†**^	Baseline^*^	**Test**^**†**^	Baseline^*^	**Test**^**†**^	Baseline^*^	**Test**^**†**^
**Mean daily count**	4.7	4.8	0.07	0.2	4.2	5.0	13.6	17.9	4.1	7.3
Standard deviation	2.2	2.1	0.3	0.5	2.1	2.5	4.0	6.0	3.0	8.0
95% Confidence Interval	4.6-4.9	4.4-5.5	0.05-0.09	0.2-0.3	3.9-4.5	4.5-5.4	13.0-14.2	16.8-19.0	3.3-4.9	5.9-8.7
**Median daily count**	5	5	0	0	4	5	13	18	3	5
**Minimum daily count**	0	1	0	0	0	1	5	5	0	0
**Maximum daily count**	15	12	2	2	10	14	27	34	15	36
**Day-of-the-week effect**	yes		no		no		no		yes	
evaluated by Kruskal-Wallis test	p = 0.007		p = 0.43		p = 0.70		p = 0.19		p = 0.04	
**Mean daily count**										
Monday	4.7		0.08		4.1		14.6		2.7	
Tuesday	4.4		0.08		4.0		12.3		4.9	
Wednesday	4.4		0.03		3.9		12.5		5.5	
Thursday	5.0		0.08		3.6		13.7		4.8	
Friday	4.5		0.12		4.8		14.6		3.4	
Saturday	4.7		0.08		4.3		13.2		5.5	
Sunday	5.4		0.05		4.6		14.4		2.0	
**Poisson CUSUM calibration**^$^										
no day-of-the-week variation			mean = 0.07		mean = 4.2		mean = 13.6			
			k = 1		k = 5		k =14			
			S_0_ = 1		S_0_ = 4		S_0_ = 10			
			h = 2		h = 7		h = 20			
day-of-the-week variation										
stratum 1	**Sunday**								**Sunday, Monday**	
	mean = 5.4								mean = 2.3	
	k = 6									
	S_0_ = 5								k = 3	
	h = 10								S_0_ = 3	
									h = 5	
stratum 2	**Monday-Saturday**								**Tuesday-Saturday**	
	mean = 4.6								mean = 4.8	
	k = 5								k = 5	
	S_0_ = 4								S_0_ = 4	
	h = 7								h = 7	

*baseline = baseline period for Poisson CUSUM calibration: EMD-AT: 1/2005-12/2008 (except December to March); EP-AT: 1/2006-12/2008 (except November to March); EP-BE: 3/2009-8/2009); ED-BE: 3/2009-8/2009; ED-ES: 3/2009-8/2009.

^†^test = test period for validity and timeliness assessment; covers the autumn winter wave of pandemic A(H1N1) influenza in 2009 (week 36/2009 to week 52/2009).

^**$**^Poisson CUSUM calibration: mean = baseline mean; k = reference value; S_0_ = head start value; h = threshold value.

We accounted for significant day-of-the-week variation with a stratified application of the Poisson CUSUM algorithm. If a day-of-the-week variation was evident, the Poisson CUSUM was calibrated separately for each stratum (Table 4). This calibrated algorithm was subsequently applied on the stratum-specific days during the test period.

#### Timeliness

Three approaches were used to assess timeliness: (1) comparison of peaks in the time series of reference data and syndromic surveillance data; (2) correlation of the time series of reference data and syndromic surveillance data; (3) comparison of signals generated by the Poisson CUSUM aberration detection method in the respective EMS data source against the beginning of the pandemic as defined in the reference data [35]. Since availability of reference data was only provided on a weekly basis, EMS data was aggregated per week for peak comparison and correlation analysis.

First, the epidemic peak periods (peak week) in EMS and reference data were compared based on the times series of the data sets during week 36 to week 52 in 2009.

Second, a cross-correlation function of weekly aggregated EMS and reference data time series was calculated for the period of week 36 to week 52 in 2009 [35,36]. The cross-correlation function indicates the similarity of two time series for different time lags, and this study was interested in the time lag that maximized the cross- correlation function. A correlation was considered significant if the upper boundary of the 95% confidence limit was crossed; a significant correlation combined with a negative time lag indicated that the epidemic curve of the syndromic surveillance data source developed earlier than the curve in the reference data, whereas, a significant correlation combined with a positive time lag indicated that the epidemic curve in the syndromic surveillance data sets developed later.

Third, timeliness was assessed by comparing the first signal detected by the Poisson CUSUM algorithm in each data source against the beginning of the official pandemic period in the respective reference data source. We counted the number of days from the Monday of the first official week of the autumn/winter A(H1N1) influenza pandemic as outlined in the reference data to the first day with a signal in the respective EMS data set.[37] A second approach took into consideration the amount of time required to collect and process the reference and syndromic surveillance data (reporting delay, see Table 2). Days were counted from the day of data availability in the reference data to the day after a Poisson CUSUM signal occurred in the syndromic surveillance data sources.

#### Validity assessment based on aberration detection

Since epidemic periods were indicated weekly in the reference data and aberrations in syndromic surveillance data were indicated daily, a weekly and daily approach was applied to the sensitivity and specificity calculations to ensure a range of potential sensitivity and specificity measures.

In the weekly approach sensitivity and specificity calculations were based on true-positive and true-negative flagged weeks. A week was flagged as true-positive when an aberration was detected on at least one day in a week that belonged to the officially confirmed pandemic period in the reference data. A true-negative week was flagged when CUSUM gave no signal during a week that did not belong to the official pandemic influenza period.

In the daily approach sensitivity and specificity calculations were based on true-positive and true-negative flagged days that were in accordance with the officially pandemic or non-pandemic periods respectively. The calculations were performed similarly to the weekly validity calculations.

A false detection rate also was calculated, indicating the proportion of false-positive flagged weeks or days to all Poisson CUSUM-flagged weeks or days.

### Software

The descriptive statistics and the correlation analyses were performed with IBM SPSS Statistics Version 21.0 (IBM Corp., Armonk, New York), and the CUSUM algorithm was programmed in Microsoft Excel 2010 (Microsoft, Redmond, Washington).

## Results

The characteristics of the emergency data sets are provided for the baseline period of each data set and for the test period (week 36/2009 to week 52/2009) (Table 4). The mean daily number of cases was higher in all data sets during the test period in 2009 than during the baseline period. The daily occurrence of ILI cases was generally a rare event in EP-AT data. The baseline periods were used to determine the parameters of the Poisson CUSUM aberration detection algorithm. Day-of-the week effects were present in EMD-AT data (Sunday stratum, Monday to Saturday stratum) and the ED-ES data (Sunday to Monday stratum, Tuesday to Saturday stratum). Table 4 also presents the calibrations of the Poisson CUSUM parameters for each data set.

### Timeliness assessment

**Figure 1:**
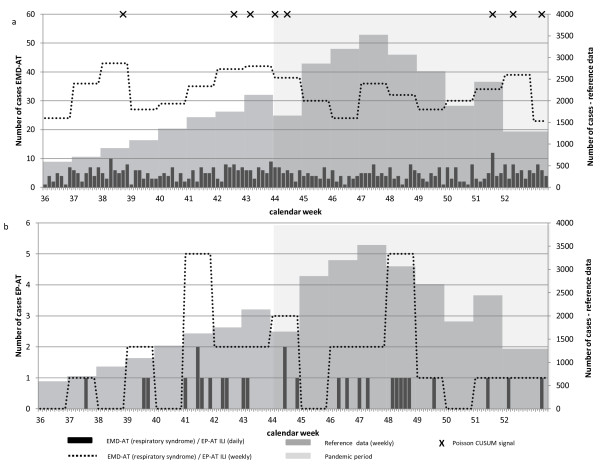
**Time series of Austrian syndromic surveillance and reference data and documentation of Poisson CUSUM signals, week 36 (30.8.) to 52 (31.12.) in 2009. a)** EMD-AT: Emergency Medical Dispatch, Tyrol, Austria. **b)** EP-AT: Emergency Physician Service (ambulances), Tyrol, Kufstein, Austria.

In Austria, the A(H1N1) reference data exhibited a peak in week 47 (Figures 1a and b). However, due to the strong variability in the EMD-AT data (Figure 1a) and the low case numbers in the EP-AT data (Figure 1b), a similar peak in these data sources could not be ascertained. Both data sets also demonstrated no significant correlation with the reference data (Table 5). Based on detected aberrations by the Poisson CUSUM algorithm, we identified one signal in EMD-AT that coincided with the beginning of the pandemic period in the reference data (Figure 1a, Table 5). Since no aberrations were identified in the EP-AT data, this approach was not viable.

**Table 5:** Results of three timeliness methods for the identification of the start of the autumn/winter wave of the A (H1N1) influenza pandemic (as reported by the reference data) with syndromic surveillance data in 2009

**Data source**	**Peak comparison**	**Cross-correlation function**	**First aberration detected by Poisson CUSUM**	
	**(weeks)**	**(weeks)**	**(days)**	
			**Without reporting delay**^**+**^	**With reporting delay**^**+**^
EMD-AT	na*	not significant	0	-6
EP-AT	na*	not significant	na*	na*
EP-BE	-1	-1	+10	+2
		0.60		
ED-BE	-1	not significant	0	-8
ED-ES	+1	+1	+19 (period 1)	+11 (period 1)
		0.89	0 (period 2)	-8 (period 2)

*na = not applicable.

^+^reporting delay refers to the time needed for collecting and processing the data.

**Figure 2:**
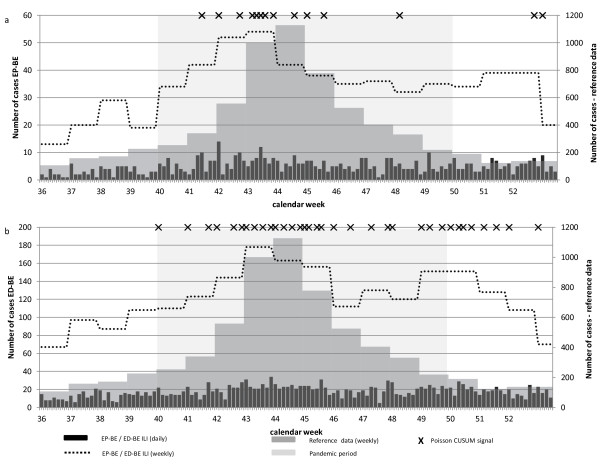
**Time series of Belgian syndromic surveillance and reference data and documentation of Poisson CUSUM signals, week 36 (30.8.) to 52 (31.12.) in 2009. a)** EP-BE: Emergency Physician Service (ambulances), Belgium. **b)** ED-BE: Emergency department, University Hospital Leuven, Flemish Brabant, Belgium.

In Belgium, the reference data peaked in week 44; however, the weekly aggregated EP-BE (Figure 2a) and ED-BE data (Figure 2b) peaked in week 43. This trend in timeliness was confirmed by the correlation analysis in the EP-BE data, which showed a significant correlation of 0.60 one week ahead of the reference data (Table 5). No statistical confirmation could be achieved in ED-BE data, which showed a non-significant correlation of 0.48 at time lag 0 (Table 5). The timeliness assessment based on the first signal generated by the Poisson CUSUM algorithm during the influenza pandemic as defined in the reference data demonstrated a slightly different picture. When taking the reporting delay of the data sets into consideration the first signal of EP-BE data was retrieved two days later than the reference data, while the signal in the ED-BE data was retrieved eight days in advance (Table 5, Figure 2b and a).

**Figure 3:**
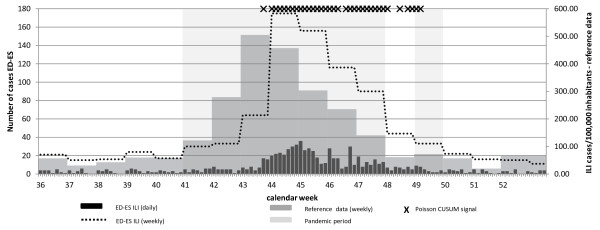
**Time series of Spanish syndromic surveillance and reference data and documentation of Poisson CUSUM signals, week 36 (30.8.) to 52 (31.12.) in 2009.** ED-ES: Emergency department, University Hospital Marqués de Valdecilla, Santander, Autonomous Region Cantabria, Spain.

The Autonomous Region of Cantabria in Spain encountered the A(H1N1) influenza pandemic peak in week 43 whereas the ED-ES data peaked one week later in week 44 (Figure 3). This observation was confirmed by a significant correlation of 0.89 at time lag +1 (Table 5). In the reference data of the Autonomous Region of Cantabria, the pandemic paused for one week (week 48) and thus two pandemic periods were available for timeliness assessment based on the Poisson CUSUM algorithm, first during week 41 to week 47 and second during week 49. This assessment showed a delayed identification of the first period (+11 days) and an earlier identification of the second period (-8 days) (Table 5).

### Validity assessment

Table 6 depicts sensitivity, specificity, and false detection rate for each data set. The number of Poisson CUSUM signals identified during the epidemic or non-epidemic periods are also presented in Table 6 and are indicated in the time series of Figures 1, 2 and 3.

**Table 6:** Sensitivity, specificity, and false detection rate of Poisson CUSUM signals for syndromic influenza surveillance during week 36 (30.8.) to week 52 (31.12.) in 2009

**Data source**	Sensitivity*%	**Specificity**^**†**^**%**	Sensitivity*%	**Specificity**^**†**^**%**	**False detection rate**^**‡**^**%**	
	**Weekly**		**Daily**		**Weekly**	**Daily**
EMD-AT	(3/9)	(5/8)	(5/67)	(53/56)	(3/6)	(3/8)
	**33.3**	**62.5**	**7.5**	**94.6**	**50.0**	**37.5**
EP-AT	(0/9)	(8/8)	(0/67)	(56/56)	(0/0)	(0/0)
	**0.0**	**100.0**	**0.0**	**100.0**		
EP-BE	(6/10)	(6/7)	(12/70)	(51/53)	(1/7)	(2/14)
	**60.0**	**85.7**	**17.1**	**96.2**	**14.3**	**14.3**
ED-BE	(10/10)	(4/7)	(26/70)	(45/53)	(3/13)	(8/34)
	**100.0**	**57.1**	**37.1**	**84.9**	**23.1**	**23.5**
ED-ES	(6/8)	(8/9)	(30/56)	(63/67)	(1/7)	(4/34)
	**75.0**	**88.9**	**53.6**	**94.0**	**14.3**	**11.8**

*Brackets: Number of weeks / days with at least one true-positive Poisson CUSUM signal in syndromic surveillance data divided by number of pandemic weeks/days according to reference data.

^†^Brackets: Number of true-negative weeks / days (no Poisson CUSUM signal) in syndromic surveillance data divided by number of weeks / days outside the pandemic period according to reference data.

^‡^Brackets: Number of weeks/days with a false-positive Poisson CUSUM signal in syndromic surveillance data divided by all Poisson CUSUM signals of syndromic surveillance data.

The ED data sets showed the strongest potential for correctly identifying the outbreak and non-outbreak periods (Table 6). The EP data sources exhibited promising results for data encompassing the entire Belgium ambulance services (EP-BE) over data for only one district in Tyrol (EP-AT). The daily measurement of sensitivity demonstrated a lower but similar pattern across the assessed data sets. The false detection rate was highest in the ED-ES and EP-BE data followed by ED-BE data.

## Discussion

The autumn/winter wave of the A(H1N1) pandemic influenza in 2009 was used as a test case to evaluate a common approach for indicator-based syndromic influenza surveillance across various European countries and EMS data sources. The highest validity was achieved by ED data from local university hospitals (ED-ES and ED-BE) followed by national data from the Belgian ambulance service (EP-BE). The timeliness assessment results indicate that detection of the beginning of the pandemic influenza occurred approximately one week sooner than in the respective reference data set in the ED-BE data and two days later in the EP-BE data. For the other data sources timeliness assessment delivered no clear results.

### Emergency department data

ED data presented the strongest validity and timeliness in this study. The only disadvantage was the delayed identification of the beginning of the autumn/winter wave in the ED-ES data. However, in this same data source the Poisson CUSUM algorithm identified the second period of pandemic influenza one week sooner than the Spanish (Autonomous Region of Cantabria) reference data.

A comparable timeliness for ED data-based syndromic influenza surveillance was identified by a study from Cowling et al., that also applied the CUSUM algorithm for aberration detection [37]. Plagianos et al. compared ILI case numbers in EDs with case numbers in ambulatory care facilities and identified a more rapid developing peak in ED data during the spring/summer wave of A(H1N1) influenza in New York in 2009 [38]. This was indicated in our study during the autumn/winter wave in the ED-BE but not in the ED-ES data.

A study on seasonal influenza after the A(H1N1) influenza pandemic in 2009, which was also based on ED-ES data, indicates that the baseline period employed for the Poisson CUSUM calibration in this study might be inflated as a result of the summer wave of pandemic A(H1N1) influenza. A lower baseline mean derived from a clear non-influenza season led to identification of seasonal influenza one week earlier in 2010/2011 and to an identification at the same time as in the reference data in the 2011/2012 seasonal influenza period [39]. The same might be true for the ED-BE data since the baseline period for this data source also included the spring/summer of 2009 due to limited data availability.

The stronger correlation and validity in ED-ES data contained in this study may be influenced by two factors. First, there are differences in the ILI coding practices. While patients in the ED-BE data were categorised as ILI cases due to one single chief complaint or working diagnosis, patients included in the ED-ES data fulfilled a more specific combined-case definition comparable to the case definition of the regional sentinel surveillance system. Second, the treatment-seeking behaviour and the use of ED services may differ between the two countries, indicating a more frequent exploitation of Spanish ED services by patients with mild conditions who could have been treated in primary care facilities [40,41]. These circumstances may have improved the representation of ILI cases in the ED-ES data and led to a better correspondence of the ED-ES data to the reference data.

### Ambulance service data

We identified no studies that applied ambulance service data (EP) for syndromic influenza surveillance. While the EP-BE data exhibited validity and timeliness measures comparable to the ED data, this result could not be confirmed by the EP-AT data since low case occurrence inhibited validity and timeliness assessment. Although it would have been possible to decrease the Poisson CUSUM threshold value for the EP-AT data, which could have resulted in certain aberration detection, we decided that the value in detecting an occasional accumulation of one or two ILI cases during a high influenza season is minimal.

Explanations for the performance differences in the two EP data sources may not be routed in differences in the coding practice between the EP-AT and the EP-BE data, as the distribution of ICD codes in ILI cases was almost comparable in both data sets. The difference may be explained by the diverging size of the catchment area of each data set: while the EP-AT data covered just one district in Austria (Tyrol), the EP-BE data were available for the entire country.

### Emergency medical dispatch data

Emergency medical dispatch data (EMD-AT) indicated the beginning of the autumn/winter wave of A(H1N1) influenza earlier than shown in the reference data. However, due to strong variability in the data set, the time series of EMD-AT did not correspond to the pattern seen in the time series of the reference data. Mostashari et al. [8] and Bork et al. [9] have also used EMD data based on comparable EMD coding systems but applied aberration detection algorithms based on regression analysis to control for several influencing variables (e.g. seasonality, holidays, temperature) [8] or dynamic forecasting models [9]. They discovered a diminished false detection rate [8] but a comparable timeliness of the system for syndromic influenza surveillance [9]. Due to the high variability and background noise of the broad EMD symptom categories, which was also seen by Coory et al. [42], it is recommended to further monitor the EMD-AT data to specify and fine-tune the aberration detection algorithm.

### Limitations

In this study, the reference data were retrieved mainly from clinical sentinel surveillance, which may be subject to over-, as well as underreporting and provides no indication regarding the virus type and subtype of ILI cases. However, clinical sentinel data are regarded as the preferred source of identifying the course of the pandemic [22], which was of primary interest in this study.

Unfortunately, historical data availability of syndromic surveillance data was limited and influenced the possibilities in calculating solid Poisson CUSUM parameters. Even though it has been demonstrated that short baseline periods are not problematic for the application of the CUSUM algorithm [43], the inclusion of the pandemic spring/summer period in 2009 might have increased the baselines in the Belgian and Spanish data sets which were only available for 2009. An increased baseline subsequently inflates the Poisson CUSUM parameters (reference value *k;* threshold value *h*) and therefore may decrease the validity and timeliness assessment during the autumn/winter period. In general, fine-tuning of the CUSUM parameters is advisable [6] and, as it was demonstrated by Schrell et al. in the ED-ES data, a recalibration of the CUSUM parameters outside the pandemic period may have increased the timeliness of our approach [39].

Additionally, we encountered constraints for the validity assessment of the daily collected data caused by weekly available reference data. We attempted to solve this problem by employing a weekly and daily approach. This allowed us to formulate ranges in which sensitivity and specificity might be located, but it should be emphasized that the daily investigation was very strict and could possibly underestimate the validity measured in this study.

We applied an aberration detection algorithm that is easy to apply, but other approaches such as regression analysis are also often used [44]. In this study, we took day-of-the-week effects into consideration and attempted to ensure that baseline numbers were not affected by seasonal influenza periods. However, other approaches are available that directly control for seasonality, day-of-the-week effects and other influencing factors such as public holidays or vacation time, and may advisably be applied in the future to increase validity and timeliness [11,45]. Additionally, it seems to be worth incorporating the monitoring of age-group specific ILI cases, especially those of children, to enhance the performance of the approach [6,46]. Given the low daily case numbers of respiratory syndrome or ILI cases in the analysed data sets, however, the stratification in age groups in this case may not lead to valid results. A weekly analysis may be possible and may solve the issue of too low case numbers [46]. For the identification of public health-relevant aberrations in EMS data, future work should also focus on the definition of alert criteria, for example, a definition of the number of consecutive days with significant aberrations in case numbers that lead to a response decision [39,47].

## Conclusion

In our study, data from emergency departments, along with data from the ambulance service covering significant catchment areas exhibited the most favourable performance in terms of validity and timeliness for syndromic influenza surveillance during the autumn/winter wave of the A(H1N1) influenza pandemic in 2009. It could be demonstrated that diverse European routine EMS data sources could be used in a common syndromic surveillance approach to gain information on sudden or out-of-season health threats. However, the individual determination of aberration detection parameters per data set is required to adjust the algorithm to the local setting.

Data from European EMS providers can support public health decision-making since these data provide timely and readily obtainable information on mostly severe cases. This information can enhance and augment various population health information data sources during health crises or other situations in which readily available health data are necessary to identify for example the effects of changing policies. A flexible and easy-to-use syndromic surveillance approach based on EMS data may be of value in improving surveillance activities in Europe.

## Abbreviations

AMPDS: Advanced Medical Priority Dispatch System; ARI: Acute respiratory infection; CUSUM: Cumulative summation detection algorithm; EC: European Commission; ECDC: European Centre for Disease Prevention and Control; ED: Emergency Department; ED-BE: Emergency department data, University Hospital Leuven, Belgium; ED-ES: Emergency department data, University Hospital Marqués de Valdecilla, Santander, Spain; EMD: Emergency Medical Dispatch; EMD-AT: Emergency medical dispatch data Tyrol, Austria which includes the city of Innsbruck, the district of Innsbruck, and the district of Kufstein; EMS: Emergency Medical Service; EP: Ambulance services staffed with emergency physician; EP-AT: Ambulance service data Tyrol, Austria (District of Kufstein); EP-BE: Ambulance service data Belgium; FIR: Fast Initial Response; GP: General Practitioner; ICD: International Classification of Diseases; ILI: Influenza-like illness; MedISys: Medical Information System; SIDARTHa: European Commission co-funded project (European emergency data-based syndromic surveillance system).

## Competing interests

The authors declare that they have no competing interests.

## Authors’ contributions

LG, GV, JBG, NR, AZ, TK and HB were involved in the syndrome definition for each data set and design of the study. NR carried out the statistical analysis. NR, AZ, TK and HB drafted the manuscript. All authors reviewed the manuscript and approved the final version.

## Pre-publication history

The pre-publication history for this paper can be accessed here:

http://www.biomedcentral.com/1471-2458/13/905/prepub
